# Distraction of Symbolic Behavior in Regular Classrooms

**DOI:** 10.3389/fpsyg.2012.00521

**Published:** 2012-11-23

**Authors:** Stefan Billinger

**Affiliations:** ^1^Swedish Institute for Disability Research, Örebro UniversityÖrebro, Sweden

**Keywords:** symbolic behavior, stimulus equivalence, matching-to-sample, classroom setting, analog observation, inclusion

## Abstract

The purpose of the present study is to develop more precise methods to explore the interaction between contextual factors in teacher instructions in regular classroom settings and students’ abilities to use symbolic information in the instruction. The ability to easily show symbolic behavior could be expected to influence student’s capacity to be active and participate. The present study examines distraction in students’ shifts from the use of “non-symbolic” to “symbolic” behavior in regular classroom settings. The 53 students (29 boys and 24 girls), ages 11–13 years old, who participated in the study were from three classes in the same Swedish compulsory regular school. Based on their test performances in a previous study, 25 students (47%) were defined as showing symbolic behavior (symbolic), and 28 students (53%) as not showing it (non-symbolic). In the present study, new test trials with distractors were added. Students from both the symbolic and non-symbolic groups scored significantly fewer correct answers on the post-training test trials with distraction stimuli (*p* < 0.05) than in post-training test trials without distraction. In the post-training test trials with competing arbitrary distractors, both groups were distracted significantly more than in the post-training test trials with competing non-arbitrary distractors (*p* < 0.05). The results indicate that a relatively easily administered and socially acceptable procedure seems to give observational data about variations in students’ symbolic behavior in relation to contextual factors in regular classroom. The main conclusion to be drawn from the results is that the observational procedure used in this study seems to have a potential to be used to explore the interaction between contextual factors and more complex student behavior such as cognition and the pragmatic use of language in regular classroom.

## Introduction

An important issue in education is the inclusion of students with disabilities with their non-disabled peers in settings that resemble as closely as possible the general educational program, while also meeting their special needs (Individuals with Disabilities Education Improvement Act, 2004; European Agency for Development in Special Needs Education, 2011). One of the most significant determents of inclusion success (Avramidis and Norwich, 2002) is the teacher’s attitudes toward inclusion. Teachers who report having support expressed more positive attitudes toward inclusion (Jerlinder et al., 2010). But to succeed teachers report the need for additional planning time, training, and specialized instructional materials (Cook et al., 2000), which may be seen as contextual factors in the students’ environment. The international classification of functioning, disability, and health for children and youth language (ICF-CY, World Health Organization, 2007) makes clear the need for more precise knowledge about the influence of contextual factors influence students’ abilities to be active and participate in learning activities (Simeonsson et al., 2008).

Previous research has established a strong connection exists between teacher-directed instructions and student achievement and learning behavior in regular classroom settings (Gettinger and Stoiber, 2009). (This study used a definition of the word *instruction* more in line with the British-English definition “something someone tells you to do” rather than an American-English definition of the word “the act of teaching someone how to do something”; www.dictionary/cambridge.org. The American-English definition refers to a more complex behavioral process that happens over a longer time than what is in focus in the study). The aim of the study is to develop methods that capture qualities in short teacher instructions that help all students to use the information in instructions in new and creative ways. In most regular classrooms teachers probably gives short instructions many time every day, telling the students what to do or presenting information about a thing, event, or phenomena. For students at risk instructions are a contextual factor that probably have a great impact on their’ ability to be active and participate.

Literature that provides teachers with recommendations concerning instructions often base their recommendations for working with students who show difficulties following instructions in regular classrooms on the students’ diagnosis or defined disability (e.g., Heward, 2009; Mastropieri and Scruggs, 2010). To be able to apply the recommendations correctly, the teacher needs to know the student’s diagnosis or defined disability. Knowing the definition of a student’s specific diagnosis probably makes a desirable difference in that it guides the schools to act in accordance with to the documented desirable effects (e.g., Silverman and Weinfeld, 2007; Silverman et al., 2009). However, there is a debate whether it is necessary to define so many students as “different,” as happens today, to help them function in school (Hjörne, 2004; Florian and McLaughlin, 2008).

Perhaps if teachers had more precise key principles to guide them in how to adjust their short instructions in regular classrooms, more students would be able to be active and participate without having to first be defined as medically different. In identifying the things teachers should do or not do to maximize learning for all students, Mastropieri and Scruggs (2010) highlight key principals for promoting clarity in teachers’ presentations (p. 135). This study was done to examine an observational procedure that may help provide such key principles because it may have the capacity to capture the functional interaction between short instructions in regular classroom and students’ abilities to show symbolic behavior based on the instruction. From a more traditional cognitive perspective, the procedure may be seen as having the potential to capture individual differences between students’ capabilities to comprehend some aspects of short teacher instructions in regular classroom settings. Analog methods may provide opportunities to simplify and reduce complex behavior constructs and observe isolated features of more global behavioral repertoires in a more manageable assessing arrangement. Analog assessment procedures appear to have some utility in identifying children who may benefit from specialized interventions (Hintze et al., 2000). Analog behavioral observation refers to observations in an environment that is designed to increase the opportunity to observe clinically important behavior and interaction (Haynes, 2001). In the present study, indirectly trained student behavior after a short training procedure in classroom are observed as an analog for the use of abstract symbolic functions of instructions in regular teaching situations.

In regular school classroom, the focus of the conversation is mostly directed by the teacher and is be full of information about subjects and things that the student may have had no prior direct experience of. The development of the ability to use symbols is a central and fundamental ability for more complex human cognitive development and one of the keys for understanding human behavior (Deacon, 1997; Carroll, 1999; Hayes et al., 2001a; Harley, 2008). Wilkinson and McIlvane (2001) argue that stimulus equivalence methodology may offer a precise way to operationalize symbolic behavior. It allows for specification of when a student shifts from the use of “non-symbolic” to “symbolic” behavior, in other words, when the student lets go of context-dependent behavior and shifts to context-free behavior.

When the teacher introduces a new concept to the students, a concept some students may not have any prior experience of, the teacher likely gives short instructions to help the students understand the meaning of the concept. The teacher may give just a few examples of situations how the concept is used and can be used, a scenario that is probably frequent in regular classrooms. If the students cannot easily use the information in the teacher instruction, beyond the few examples the teacher presented, then the students will likely be less able to be active in the learning activity. If, however, the teacher presents the information such that the students easily could act on never before experienced stimuli relations within the information (symbolic behavior), then the students likely would have greater opportunities to be active during class. The focus of this present study is to develop observational methods that more precisely capture qualities in such short teacher instructions, qualities that may make a difference to whether the students may show symbolic behavior or not. In the present study symbolic behavior is defined as something the student do when the student act as if he or she can see arbitrary relations between stimuli he or she never experienced directly before, but are able to derive from the few example of relations that are given to all students in the procedure. The procedure provides a more precise way to operationalize symbolic behavior then just referring to the use of words as symbols. Relational frame theory (Hayes et al., 2001a) defines such a behavior as derived relational responding (DRR).

By using a matching-to-sample procedure (a stimulus equivalence methodology) the intention is to observe student response as a type of derived relational response. The procedure ensures that the students show responses that are not a result of direct experiences or that can be traced to a history of reinforcement. DRR is used as a way to observe what is in this study is defined as the shift from non-symbolic to symbolic student behavior.

Research with a focus on DRR in connection to students with autism and other developmental disabilities has shown promising results regarding students’ desired functional development (Rehfeldt and Barnes-Holmes, 2009). The research reports indicate that the ability to see and respond to derived stimulus relations differs between children and within children. And it seems as if this ability is a learned capacity and possible to influence by training and adaptation of the environment. Rehfeldt and Barnes-Holmes (2009) show that the research on DRR and education so far has not focused on the interaction between contextual factors in regular classroom environment and the student’s performances. The observational procedure used in this study may have the potential to produce more knowledge on how to classify contextual factors and adapt the regular classroom to meet variations among students. This kind of observational procedures may have the potential to provide meaningful information about the interaction between frequently occurring contextual events in regular classrooms and more precisely descriptions of shifts in a special type of student behavior, which is hard to observe in everyday life and realistic settings. The procedure is easy to administer and seems to be readily accepted by students, teacher, and parents; it does, not interfere too much in regular school activity.

In a previous experiment, Billinger and Norlander (2011) showed that the observational procedure used in this study seems to have the potential to produce data about the interaction between a presented “instruction” and individual differences between students showing “non-symbolic” and “symbolic” behavior. The participants in this study also took part in the Billinger and Norlander (2011) study. Some pre-test results from the 2011 study (the results) are being used in this study. The same basic stimulus equivalence methodology, a matching-to-sample procedure (Green and Saunders, 1998) has been used also but in this study it has been used to examine distraction in shifting from non-symbolic to symbolic behavior.

In the present study we used two different distractors: stimuli that have a non-arbitrary relation to the sample stimulus the students are to match comparison stimuli to, and stimuli that have an arbitrary relation to the sample stimulus. Non-arbitrary distracting stimulus is stimuli that are physically similar in some aspects, for, both stimuli might be red or colored (red and green). In some cases, stimuli can be shapes, even though they may be different shapes. An example of an arbitrary distracting stimulus for an English-speaking person is the word “square” in relation to a visual shape of a square. RFT advocates that we, as humans, generally have difficulties letting go of previously learned arbitrary relations (Törneke, 2010). The flexibility, the ability, to “let go” of a previous learned symbolic relations would be of a great advantage in contexts such as the regular classrooms. In everyday life it is difficult to determine whether the stimulus functions guiding a student’s behavior were established directly or through derived relational responses because arbitrary and non-arbitrary relations nearly always affect human behavior in complex combinations (Törneke, 2010). The observational procedure used in this study might make it possible to more precisely evaluate whether the students’ behavior is guided by direct or indirect stimulus relations in the interaction with contextual factors in short instructions in regular classrooms.

Based on the previous research, it was hypothesized that students who had previously shown a capacity to quickly respond with symbolic behavior, would show less distraction than students who had not shown the same capacity. It was also hypothesized that students would show significantly more distraction in test trials that used a “competing” comparison stimulus with a previously experienced arbitrary relation to the sample stimulus, than in trials that used a comparison stimulus with a non-arbitrary relation to the sample stimulus.

## Materials and Methods

### Participants

The participants in this study also participated in the Billinger and Norlander (2011) study. Initially 70 students had participated, but only 53 students (29 boys and 24 girls) followed all procedures and completed the study. The 53 scored all training trials correctly (18 of 18) at the end of previous training sequences, and on that basis were included in the current experiment. They ranged in age from 11 to 13 years of age and were recruited from three different classes in the same compulsory regular school in Sweden. The target school had approximately 200 students ranging in age from 6 to 13 and is regarded as a normal school without any particular differential characteristics. The three classes were also seen as ordinary.

### Materials

#### Paper pad

At the beginning of each session in the experiment, each student was given a paper pad that had a page for each training and test trial. Every second page was colored (to help the test leader see when all students had made their responses in that trial and turned to the next page). Each page had the trial number in small numerals in the bottom right corner. Each page had three printed X crosses (XXX), representing the row of three comparison stimuli shown to the class. The children were instructed to circle the cross that represented the comparison stimulus they thought matched the sample stimulus.

#### Visual stimuli

**Figure 1:**
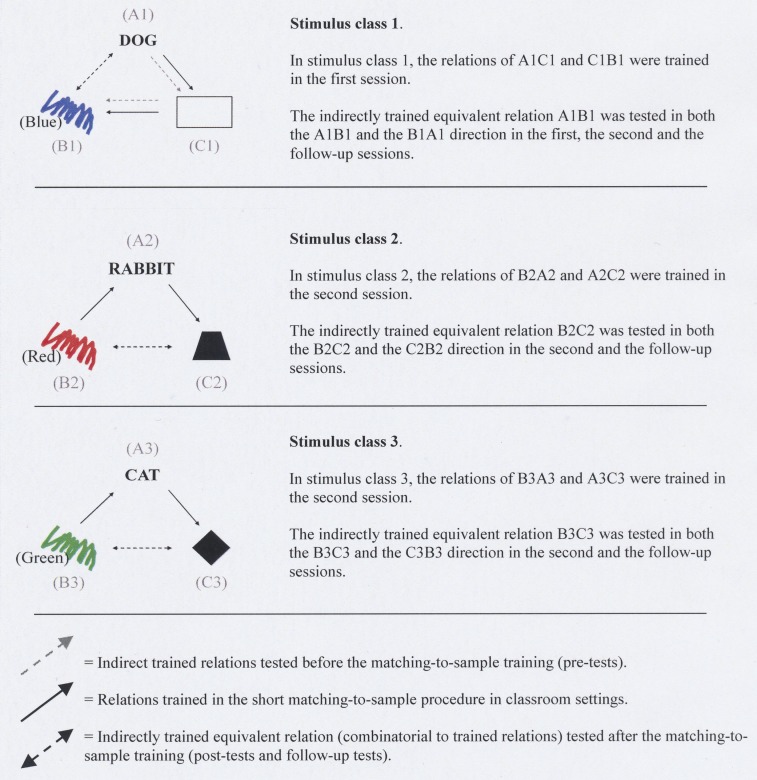
**The three stimulus classes (1, 2, and 3) and the stimulus relations that had been pre-tested, trained, post-tested before the current study**. The words (Blue), (Red), (Green), in the trial examples illustrated, were not visible to the students. The same applies to Figure 2. From Billinger and Norlander (2011), Copyright (2011) by Billinger and Norlander. Reprinted by permission.

In the previous experiment (Billinger and Norlander, 2011), nine main visual stimuli had been used in the sequence to identify the students who showed symbolic behavior (see Figure 1). To facilitate explanation, each stimulus had been designated with an alphanumeric label (e.g., A1, B2, C3), which were not shown to the students. The stimuli were images of familiar things such as Swedish words for “dog” (A1), “cat” (A2), “rabbit” (A3), and colored patches (blue, B1; red, B2; green, B3), and finally geometric forms (rectangle, C1; rhombus, C2; trapezoid, C3).

In all matching-to-sample trials (the previous training and test trials, and the test trials for this study) the visual stimuli were projected onto a silver screen that was visible to all students at the same time. The images of the stimuli were projected from a PowerPoint file.

**Figure 2:**
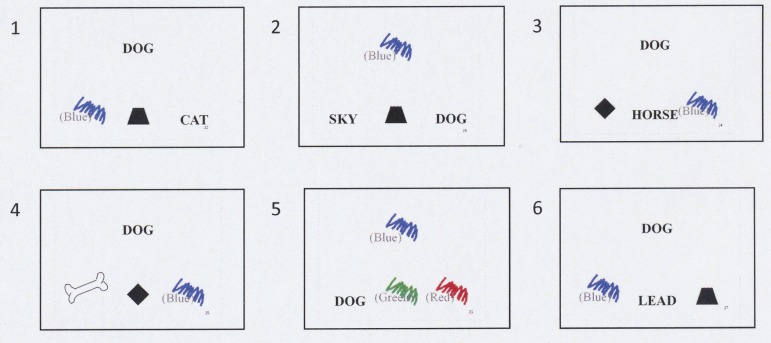
**The six test trial used to test distraction effects**.

In this experiment, four more stimuli were used: a picture of a dog bone, and the Swedish words “sky,” “horse,” and “lead” (see Figure 2).

##### Design

Six test trials were conducted find out whether the two groups of students would differ in their response as if they were distracted in their symbolic behavior and whether they would respond as if they were more distracted by arbitrary stimuli than non-arbitrary stimuli. The reason behind the design was to be able to determine whether students would score as if they were capable of “seeing” the derived (indirect) equivalent stimulus relations after a fixed number of training trials and in competition with a distracting comparison stimulus. Green and Saunders (1998) “Minimal Training and Test Trial Types for a Prototypic Stimulus Equivalence Experiment” (p. 241) was used as the basic model for the training procedure (Billinger and Norlander, 2011) and the test procedure in this study. This model was chosen due to its minimal design, which was believed to increase the practical use and social acceptability of the procedure.

A mixed two-way ANOVA was used to evaluate the effects the two test conditions had on students’ test trial scores. The within-subject factor was distracting test conditions, that is the number of correct scores on test trials with either non-arbitrary distractors or arbitrary distractors. The between-subject factor was whether student was defined as showing symbolic behavior.

#### Procedure

All students participated of their own free will and could leave the study at any time. The students’ parents were informed of the study by letter and had to give their written approval of their child’s participation in the study. The present experiment was conducted consistent with the ethical rules and considerations within the *national Swedish act* “*Ethical Review of Research Involving Humans* (*2003*:*460*).” The sessions were conducted in the student’s regular class and classroom.

The main structure of the procedure was the same in all sessions and took 10–20 min to administer. The students sat in their regular seats taking notes, and the experimenter stood in front of the class showing the slides. Both the experimenter and the teacher were present, encouraging the students not to whisper to each other but to work independently.

The six test trials were conducted immediately after the students had been trained and tested. In the formation of three 3-member equivalence classes (A1-B1-C1, A2-B2-C2, A3-B3-C3, see Figure 1), two of the relations between stimuli in each stimulus class were trained and one relation was tested. The three stimulus classes had been randomly defined. The stimuli within each stimulus class are related to each other as same as and this had been randomly determined before the experiment. Thus, all three arbitrary stimuli relations in each stimulus class (e.g., A1B1, A1C1, B1C1, i.e., DOG-blue patch, DOG-rectangle, blue-patch-rectangle) were random, and none of the students could have had prior knowledge of which stimuli would relate to each other as same as within each stimulus class. All trials in both experiments started such that the sample stimulus [e.g., the word DOG (A1)] was visible in the upper center part of the screen (see Figure 2). After that three comparison stimuli were visible in a row in the lower part of the screen [e.g., a rectangle (C1), a rhombus, (C2), and a trapezoid, (C3)]. None of the comparison stimulus had any obvious relation to the sample stimulus. The student’s task was to choose one of the three comparison stimuli to match the sample stimulus.

The six test trials used in the present study differed from those in the previous study. In that study the student had shown they had learned to respond correctly in training trials that were directly trained. Immediately after the training trial, the students were told which comparison stimulus was correct. In the test trials, the students were tested on stimulus relation between the stimuli not directly trained.

In the experiment done for this study, four new stimuli were used, as well as the nine stimuli used in the previous study (see Figure 2). This study tested indirectly (derived) stimulus relations in “competition” with comparison stimuli. These comparison stimuli have a familiar non-arbitrary and/or arbitrary relation to the sample stimulus. Every student was expected to have had a lot of experience of these stimulus relations before the experiment. Test trial numbers 1, 3, and 5 were designed to test the indirectly trained relation between A1B1/B1A1 in competition with non-arbitrary stimulus relations (see Figure 2). For example, in test trial 1 the comparison stimulus, “a blue patch” (B1), is the correct choice. The sample stimulus “DOG” (A1) has a derived “same as” relation to B1 in the experimental context. But, the students were expected to see the physical non-arbitrary relation between the comparison stimulus “CAT” and the sample stimulus “DOG.” The stimulus relation between “CAT” and “DOG” is here seen as a primarily non-arbitrary physical relation that will compete in this context with the indirectly trained (derived) arbitrary stimulus relation between A1 and B1. The A1B1 stimulus relation might be seen as the correct “same as” relation to respond to in this present classroom context. The stimulus relation A1 and B1 might be seen as a more decontextualized stimulus relation, more dependent on association to a previous experience in an earlier context and perhaps harder to see than the contextual more explicit stimulus relation between two present visible words. The assumption is that some students might respond as if they were distracted on what is the correct “same as” relation.

Test trial numbers 2, 4, and 6 were designed to test the indirectly trained relation between A1B1/B1A1 in competition with arbitrary stimulus relations (see Figure 2). For example, in test trial number 4 the comparison stimulus “a blue patch” (B1) is the correct choice. The students were expected to see the non-physical but arbitrary relation between the comparison stimulus “an image of a dog bone” and the sample stimulus “DOG.” The stimulus relation between “an image of a dog bone” and “DOG” is here seen as a primarily arbitrary relation that will compete in this context with the indirectly trained (derived) arbitrary stimulus relation between A1 and B1. The A1B1 stimulus relation might be seen as having the correct “same as” relation to respond to in this classroom context. The stimulus relation A1 and B1 might be seen as a more decontextualized stimulus relation, more dependent on association to previous experience in an earlier context and perhaps harder to see than the contextual more explicit stimulus relation between two present visible stimuli the student probably has experienced been meaningful related to each other in a lot of contexts.

After the training and test trials were complete, the experimenter and the teacher collected each student’s paper pad. The experimenter later identified each student response as either correct or not correct. A student response was defined as correct when the correct “X” was clearly marked. Unclear responses (e.g., a student had marked more than one option) were defined as incorrect.

## Results

In the post-test trials in the previous experiment (Billinger and Norlander, 2011), 28 (53%, 15 boys, 13 girls) of the 53 students had not shown symbolic behavior (non-symbolic), while 25 (47%, 14 boys, 11 girls) had shown symbolic behavior. These 53 students were in the current experiment and were grouped in a non-symbolic student group and a symbolic student group. In the present study new post-training test trials with distractors were added.

A mixed two-way ANOVA was performed with distraction (non-arbitrary or arbitrary distractors) as the within-subject factor and symbolic behavior as the between-subject factor (non-symbolic and symbolic). The dependent variable was the number of correct scores on test trials. Data analyses showed there was a significant impact of distraction [*F* (1, 51) = 15.58, *p* < 0.001]. For means and standard deviations see Table 1. *Post hoc* tests (Pair-Samples *t*-test, 5% level) showed significantly fewer correct test scores when the distraction was arbitrary. That result indicates that the distraction, whether non-arbitrary or arbitrary, had a significant impact on the students’ abilities to show symbolic behavior. Arbitrary distractors seem to distract more profoundly. *Post hoc* data (Pair-Samples *t*-test, 5% level) show that when the tests trial includes distractors (non-arbitrary or arbitrary) the number of correct scores drops significantly for both sub groups, compared to post-test trials without distractors.

**Table 1:** **Mean values (*M*) and standard deviations (SD) for scorings concerning test trials with different test conditions**.

Test conditions	Non-symbolic			Symbolic		
	*n*	*M*	SD	*n*	*M*	SD
**PRE TRAINING TEST**						
No distracter	28	0.96	0.92	25	0.92	1.12
**POST-TRAINING TEST**						
Non-arbitrary distracter	28	1.54	1.14	25	1.68¤	1.28
Arbitrary distracter	28	0.93¤	1.30	25	1.32¤	1.25
No distracter	28	2.20¤	0.74	25	2.67*¤	0.40

*Indicates when students with symbolic behavior scored significantly higher when comparing to students with non-symbolic behavior.

*¤Indicates a significant difference from the tabulated value above*.

The data show no significant impact on the between-subject factor Symbolic Behavior. *Post hoc* tests (Independent-Samples *t*-test, 5% level) showed significant difference between the two sub groups on post-test trial without distractors but not on post-test trials with distractors. Finally, the data show no Distraction × Symbolic behavior interaction. Although *post hoc* tests (Pair-Samples *t*-test, 5% level) show that non-symbolic students scored as though the training had no effect when there were distractors in the test trials, whereas symbolic students scored significantly more correct answers on test trials with non-arbitrary distractors than on the pre-test trials. Arbitrary distractors distracted more significantly then non-arbitrary distractors for both non-symbolic and symbolic students. The present data does not clearly predict how much distraction we might expect from a student, in relation to how quickly he or she previously has been shown symbolic behavior or whether the distractor is arbitrary or not. However, the result indicates that the quality aspects of the distraction had a significant impact on the students’ capabilities to show symbolic behavior.

In the previous experiment (Billinger and Norlander, 2011), we concluded that low scores on pre-test and high scores on post-test indicated that the students had learned to respond to (to see) derived (indirect) stimulus relations within the stimulus class. The students’ pre-test scores indicate they had no previous experience of the stimulus relations before the short training sequence. Then, just a few minutes later, in the post-test trials they responded as though they could see them. The overall results from the previous study indicated that the procedure has a potential to differentiate between directly learned student behaviors and indirectly symbolic students’ behaviors in classroom settings. The overall results from the current experiment indicate that the procedure also seems to be able to produce data sensitive to the different factors that may distract students’ behavior in regular classrooms contexts.

## Discussion

The results of the study indicate that the systematic use of contextual distractors in regular classrooms seems to systematically distract students’ symbolic behavior. Both the students who had shown a capacity to quickly respond with symbolic behavior and those who had not, scored as if they were distracted. And all the students showed significant more distraction in test trials with a “competing” comparison stimulus that had a previously experienced arbitrary relation to the sample stimulus, than in tests with a comparison stimulus that had a non-arbitrary relation to the sample stimulus.

As in the Billinger and Norlander (2011) study the students’ use of symbolic behavior seems to vary depending on contextual factors. In the Billinger and Norlander (2011) study the students showed variations in the symbolic behavior in interaction with the same contextual factors presented in their regular classrooms. If the ability to quickly see symbolic relations is important for students’ capacity to be active and participate in a learning activity that variation could be important. No previous study had observed the interaction between contextual factors and complex student behavior such as symbolic behavior in regular classrooms. When we changed some contextual factors, we added stimuli we suspected would distract the students from guiding themselves to matching which stimulus that belonged to which. All the students scored as if they were distracted. They scored as though the stimulus relation that would be the correct one to derive from the information in the present context did not guide them.

The first hypothesis in this study was that the “non-symbolic students” would score as if they were more distracted than the “symbolic students,” but the results show that they did not. These results may be seen as an indication that even if a student has learned to quickly “see” derived stimulus relation in new information, stimulus relation previously directly reinforced in known contexts still guides their response. From that perspective, the results seem reasonable and it is logical that a meaningless stimulus relation should not “win” over a meaningful stimulus relation even if they see the new derived relation in the present context.

Three questions are of special interest in connection with further studies: (1) Is a type of DRR being observed among the students? (2) If it is DRR, does the observed variation correlate to other important student responses, as the theoretical assumption suggests? (3) Would similar variations be observed in other situations and/or with other students in interaction with the same contextual factors?

In regard to the first question, according to the definition cited in the introduction, it is reasonable to argue that a type of DRR was observed because the study used a procedure based on a basic matching-to-sample procedure (Green and Saunders, 1998). The procedure ensures “that responding is not the result of direct experiences that can be traced to a history of reinforcement involving the stimuli in question or to formal similarity between or among the stimuli” (Moore, 2009. p. 33).

In regard to the second question, the fundamental issue is whether the variation observed with the procedure is meaningful in relation to the students’ abilities to be active and participate after being given short teacher instructions in real life settings. We are aiming to gain the knowledge needed to create a short training sequence as a contextual factor that would help for the students in the study not to be distracted. An assumption is that in the long run, if teachers use the knowledge in their short instructions, it could help make the same student significantly more active and enable them to participate more in real learning activities. Further studies are needed to explore the relation between student responses in the procedure and their activity and participation in learning activity in everyday life.

The third question is whether the results are generalizable. Based on the theoretical assumption is that DRR is a learned response (Hayes et al., 2001b), a certain stability could be expected in how different individuals responded to the same stimuli in a different setting. Further studies are needed to explore how students respond using the present procedure in one setting would respond in other settings and/or explore how well different students would respond in the same setting.

The results indicate the procedure might be said to have the potential to identify instructional situations in which some student might be at risk of being distracted. While systems have been developed for observation in educational setting (e.g., Greenwood et al., 2000) none we found has focused on the interaction between instructions and students’ abilities to show symbolic behavior in regular classrooms. In order to move forward in the inclusion process, there is a need to observe the interaction between contextual factors influence on students’ responses in regular classrooms. My main conclusion from the present result in relation to previous results is that the observational procedure used in this study seems to have a potential to be used to explore the interaction between contextual factors and more complex student behavior such as cognition and the pragmatic use of language in regular classroom.

## Conflict of Interest Statement

The author declares that the research was conducted in the absence of any commercial or financial relationships that could be construed as a potential conflict of interest.
